# Bladder dysfunction in human T cell lymphotropic virus infection: A prospective cohort study

**DOI:** 10.1371/journal.pntd.0009772

**Published:** 2022-01-14

**Authors:** José Abraão Carneiro Neto, Cássius José Vitor de Oliveira, Sheila Nunes Ferraz, Mariele Guerra, Lívia Alves Oliveira, Lúcia Passos, Edgar M. Carvalho, Paulo Novis Rocha

**Affiliations:** 1 Post Graduate Program of Health Sciences, Federal University of Bahia, Salvador, Bahia, Brazil; 2 Immunology Service of University Hospital Professor Edgard Santos, Federal University of Bahia, Salvador, Bahia, Brazil; 3 Gonçalo Moniz Institute, Fiocruz, Salvador, Bahia, Brazil; 4 National Institute of Science and Technology of Tropical Diseases, Salvador, Bahia, Brazil; 5 Nephrology Service of University Hospital Professor Edgard Santos, Federal University of Bahia, Salvador, Bahia, Brazil; Beijing Children’s Hospital, Capital Medical University, CHINA

## Abstract

**Background:**

While bladder dysfunction is observed in the majority of patients with human T cell lymphotropic virus type 1 (HTLV-1)-associated myelopathy (HAM), it is also observed in patients who do not fulfill all diagnostic criteria for HAM. These patients are classified as having possible or probable HAM/TSP. However, it remains unclear whether the severity and progression of bladder dysfunction occurs similarly between these two groups.

**Objective:**

Compare the severity and evolution of bladder dysfunction in HTLV-1-infected patients with possible and definite HAM/TSP.

**Methods:**

The present prospective cohort study followed 90 HTLV-1 patients with possible HAM/TSP and 84 with definite HAM/TSP between April 2011 and February 2019. Bladder dysfunction was evaluated by bladder diary, overactive bladder symptoms scores (OABSS) and urodynamic studies. Bladder dysfunction progression was defined as the need for clean self-intermittent catheterization (CIC).

**Results:**

At baseline, nocturia, urgency and OABSS scores were worse in definite compared to possible HAM/TSP patients. The main urodynamic finding was detrusor overactivity, present in 77.8% of the patients with definite HAM/TSP versus 58.7% of those with possible HAM/TSP (P = 0.05). Upon study conclusion, the cumulative frequency of patients requiring CIC increased in both groups, from 2 to 6 in possible HAM/TSP and from 28 to 44 in definite HAM/TSP patients. The estimated time to need for CIC was 6.7 years (95%CI 6.5–7.0) in the possible HAM/TSP group compared to 5.5 years (95%CI 4.8–6.1) in the definite HAM/TSP group.

**Conclusions:**

Although both groups showed similarities in bladder dysfunction and tended to progress to requiring CIC over time, patients with possible HAM/TSP presented less severe manifestations at baseline and progressed more slowly than those with definite HAM/TSP.

## Author summary

The present prospective cohort study followed 90 HTLV-1 patients with possible HAM/TSP and 84 with definite HAM/TSP between April 2011 and February 2019. Bladder dysfunction was evaluated by bladder diary, overactive bladder symptoms scores (OABSS) and urodynamic studies. Bladder dysfunction progression was defined as the need for clean self-intermittent catheterization (CIC). At baseline, nocturia, urgency and OABSS scores were worse in definite compared to possible HAM/TSP patients. The main urodynamic finding was detrusor overactivity, present in 77.8% of the patients with definite HM/TSP versus 58.7% of those with possible HAM/TSP (P = 0.05). Upon study conclusion, the cumulative frequency of patients requiring CIC increased in both groups, from 2 to 6 in possible HAM/TSP and from 28 to 44 in definite HAM/TSP patients. The estimated time to need for CIC was 6.7 years (95%CI 6.5–7.0) in the possible HAM/TSP group compared to 5.5 years (95%CI 4.8–6.1) in the definite HAM/TSP group. Although both groups showed similarities in bladder dysfunction and tended to progress to requiring CIC over time, patients with possible HAM/TSP presented less severe manifestations at baseline and progressed more slowly than those with definite HAM/TSP.

## Introduction

The human T cell lymphotropic virus type 1 (HTLV-1) infects 5–10 million people worldwide [1–3]. South and Central America, Japan, Romania, West Africa and Australia have been identified as endemic areas [4]. HTLV-1 is a retrovirus that predominantly infects T lymphocytes [5]. It is the causal agent of HTLV-1-associated myelopathy/tropical spastic paraparesis (HAM/TSP) and adult T-cell Lymphoma/Leukemia [6]. HTLV-1 infection is considered neglected because most affected patients come from low and middle income countries and also due to low morbidity, as less than 5% of patients develop severe forms of disease [7]. However, it has recently been established that a large percentage of HTLV-1-infected subjects may present other diseases or clinical manifestations related to the infection, including sicca syndrome, periodontal disease, HTLV-1-associated arthropathy, uveitis, pulmonary disease and urinary and/or sexual dysfunction [8–11]. While symptoms of overactive bladder (OAB), characterized by nocturia, increased daytime urinary frequency (DUF) and urgency with or without incontinence, are observed in virtually all patients diagnosed with definite HAM/TSP, up to 37% of HTLV-1-infected subjects who do not meet the clinical criteria for definite HAM/TSP also present OAB [12,13]. Individuals who suffer from urinary dysfunction in the absence of motor disability are considered to have possible or probable HAM/TSP [14].

Urinary dysfunction in affected individuals decreases quality of life (QoL) [12,15], increases the risk of urinary tract infection [16] and may lead to kidney injury [17]. Patients with possible and definite HAM/TSP who suffer from urinary manifestations also present higher proviral load and produce comparatively higher levels of proinflammatory cytokines than HTLV-1 carriers without myelopathy [18]. Data in the literature are inconclusive as to whether bladder dysfunction evolves similarly in patients with possible and definite HAM/TSP. Nocturia usually is the initial urinary manifestation most commonly observed in HTLV-1-infected subjects. Thereafter, some patients develop increased DUF, urgency and incontinence; eventually, this may progress to bladder atony/areflexia [19]. However, this clinical course has not been conclusively documented due to a lack of prospective studies evaluating urinary tract dysfunction in association with HTLV-1. Indeed, it is not known if possible HAM/TSP patients with urological symptoms alone suffer from an isolated clinical entity, or whether these individuals will progress to definite HAM/TSP. The present study endeavored to characterize baseline urological manifestations and compare the evolution of bladder dysfunction in a cohort of patients with possible and definite HAM/TSP.

## Materials and methods

### Ethics statement

All enrolled patients signed an informed consent form, and the study was approved by the Ethics Committee of Federal University of Bahia. Since 2011, patients with storage and voiding symptoms have been recruited to the cohort by attending urologists. Demographic, clinical, neurological and LUTS data were systematically registered in a database. After ruling out urinary tract infection, urodynamic studies were performed in accordance with International Continence Society (ICS) recommendations [20].

### Study design and patients

This prospective cohort study was conducted at an outpatient clinic specialized in the care of patients with HTLV-I, located in a tertiary university hospital in Salvador, Bahia-Brazil. The main sources of patient referral to this clinic are blood banks and other specialty clinics within our hospital complex. All patients with HTLV-1 infection detected by ELISA had confirmation by Western blot. For this study, we screened all HTLV-1 patients that presented between April 2011 and February 2019 for the presence bladder dysfunction. All patients with lower urinary tract symptoms (LUTS) were subjected to urine cultures. Only patients with LUTS and negative urine cultures were considered eligible for inclusion. Exclusion criteria were a previous history of stroke, pelvic or neurological surgery, severe brain trauma or spinal cord injury, other myelopathy, coinfection with HIV, HBV, HCV or HTLV-2, and pregnancy.

The included patients were evaluated twice a year by voiding diary, the overactive bladder symptoms score (OABSS) questionnaire, and physical and laboratorial examinations. DUF, episodes of nocturia and/or incontinence, urinary retention and the use of treatments (anticholinergic drugs, physiotherapy, intradetrusor application of onabotulinumtoxin Type A, or bladder augmentation with catheterizable conduit) were recorded.

### Case definition

The diagnostic criteria proposed by Castro-Costa [14] were applied to determine definite HAM/TSP, characterized by an Osame motor disability scale (OMDS) score >1, difficulty or inability to walk, and spasticity. Patients with possible HAM/TSP presented OAB without gait impairment, but could have hyperreflexia and/or a positive Babinski sign [14].

### Bladder dysfunction

Bladder dysfunction was defined by the presence of storage or voiding symptoms characterizing OAB or underactive bladder, respectively [11,19,21]. OAB was defined by an urgency to void, with or without incontinence, usually accompanied by frequency and nocturia and mandatory negative urine culture [22,23]. Incomplete bladder emptying was documented by a high post-void residual volume (≥100 ml) measured by echography or by clean self-intermittent catheterization (CIC) [24]. This was considered the main finding to determine the presence of underactive bladder.

### Neurological evaluation

Patients were evaluated by a neurologist and, in addition to the OMDS, the expanded disability status scale (EDSS) was also applied.

### Urodynamic evaluation

Evaluations were carried out in accordance with the guidelines established by the American Urologic Association/Society of Urodynamics and Female Pelvic Medicine & Urogenital Reconstruction (AUA/SUFU) and ICS recommendations [20,25,26].

### Bladder dysfunction progression

The progression of bladder dysfunction was defined as the need for CIC to promote bladder emptying.

### Statistical analysis

Continuous data were expressed as mean ± standard deviation (SD) or median and interquartile range (P25 –P75). Categorical variables were described using absolute and relative frequencies. Comparisons of continuous variables between the two study groups, possible HAM/TSP and definite HAM/TSP, were performed using the Student’s t-test for independent samples or the Mann-Whitney U test. Categorical variables were compared using the chi square test. Kaplan-Meier survival analyses were employed to compare time elapsed until need for CIC, and survival curves were compared using the log-rank test. Life-table analysis was also performed to identify the number at risk, number of events and number censored in both groups at each timepoint; these data were then added to the Kaplan-Meier survival figures. We adopted a P value < 0.05 to define statistical significance. All statistical analyses were performed using the IBM Statistical Package for the Social Sciences (SPSS) Version 27.

## Results

From April 2011 to February 2019, a total of 193 adult HTLV-1 patients with LUTS and negative urine cultures were screened. Of these, 19 patients were excluded for the following reasons: 11 due to incomplete data and 8 due to coinfection with other viruses (5 with HIV, 2 with hepatitis B and C virus, and 1 with HTLV-2). Baseline clinical and demographic data on the remaining 174 patients, stratified into possible and definite HAM/TSP, are shown in Table 1.

**Table 1:** Baseline Clinical and Demographic Features of HTLV-1 Infected Patients with Possible and Definite HTLV-1 Associated Myelopathy Followed in a Cohort Study.

Variables	Possible HAM/TSP N = 90	Definite HAM/TSP N = 84	P Value
Age (mean ± SD), years	51.3 ± 12.0	51.3 ± 13.0	1.00
Female sex (%)	70/90 (77.8)	64/84 (76.2)	0.95
Non-caucasian (%)	79/90 (87.8)	67/84 (79.8)	0.22
Education: Less than high school (%)	81/90 (90.0)	73/84 (86.9)	0.69
Married (%)	54/90 (60.0)	42/84 (50.0)	0.24
DUF (mean ± SD)	7.0 ± 2.7	8.1 ± 5.2	0.13
No episodes of urgency (mean ± SD)	2.4 ± 2.1	3.9 ± 5.1	0.02
No episodes of nocturia (mean ± SD)	3.0 ± 1.8	4.0 ± 2.4	0.00
OABSS	7.8 ± 3.7	9.8 ± 3.8	0.00
CIC (%)	2/84 (2.4)	25/82 (30.5)	0.00
OMDS (median; P25 –P75)	0.0 (0.0–0.0)	5.0 (3.0–7.0)	0.00
EDSS (median; P25 –P75)	0.0 (0.0–2.0)	5.0 (3.0–6.8)	0.00
Follow-up (mean ± SD), years	5.2 ± 1.1	5.2 ± 1.1	0.90

Abbreviations: SD: Standard deviation; EDSS: Expanded Disability Status Scale; OMDS: Osame’s Motor Disability Scale; DUF: Daytime Urinary Frequency; OABSS: Overactive Bladder Symptoms Score; CIC: Clean self-intermittent catheterization. For continuous variables, maximum missing data ranged from 5.7% for demographics (follow up) to 13% for clinical variables (OABSS).

No differences were observed among the groups regarding age, gender, self-declared skin color or marital status. The majority of patients in both groups complained of storage symptoms: increased DUF, nocturia and urgency. The mean frequency of nocturia and urgency episodes, as well as OABSS at baseline, were lower in the possible HAM/TSP group compared to the definite HAM/TSP group. The number of patients requiring CIC to promote bladder emptying was significantly higher (P<0.01) in the definite HAM/TSP group.

### Urodynamic analysis

Urodynamic studies were carried out in 92/174 HTLV-1-infected patients upon entry in the cohort (Table 2). Detrusor overactivity (DO) was the most common urodynamic finding in both groups, which tended to be more frequent in definite HAM/TSP patients. Detrusor underactivity (DU) was more common in definite compared to possible HAM/TSP patients (P<0.001). The definite HAM/TSP patients also had lower voided volume and tended to have higher post-void residual volume (PVR) as well as increased bladder filling sensation than patients with possible HAM/TSP. No significant differences were seen between the two groups regarding functional bladder capacity (FBC), compliance, bladder outlet obstruction (BOO) and maximum flow rate (Qmax) during the emptying phase of urodynamic studies.

**Table 2:** Baseline Urodynamic Findings in a cohort of 92 HTLV-1-Infected Patients; Evaluation of Urinary Manifestations in Patients with Possible and Definite HTLV-1 Associated Myelopathy.

Variables	Possible HAM/TSP N = 46	Definite HAM/TSP N = 46	P Value
Bladder filling sensation (%)			
Normal	35/46 (76.1)	21/44 (47.7)	0.004
Decreased	6/46 (13.0)	5/44 (11.4)	
Increased	5/46 (10.9)	18/44 (40.9)	
FBC—mL (mean ± SD)	353 ± 127	330 ± 151	0.33
Compliance—mL/cmH2O (mean + SD)	50.5 ± 62.2	73.3 ± 156.6	0.06
DO (% Yes)	27/46 (58.7)	35/45 (77.8)	0.05
VV—mL (mean ± SD)	265.2 ± 184.8	132.8 ± 147.7	0.02
PVR—mL (mean ± SD)	125 ± 175	191 ± 156	0.07
DU (% Yes)	6/45 (13.3)	23/45 (51.1)	0.001
BOO (% Yes)	30/44 (68.2)	21/42 (50.0)	0.08
Qmax—mL/s (mean ± SD)	15 ±15	9.5 ± 11	0.10

Abbreviations: FBC: functional bladder capacity; SD: Standard deviation; DO: detrusor overactivity; VV: voiding volume; PVR: post voiding residual volume; DU: detrusor underactivity; BOO: bladder outlet obstruction; Qmax: Maximum flow rate.

### Clinical evolution of HTLV-1 infected patients

**Fig 1:**
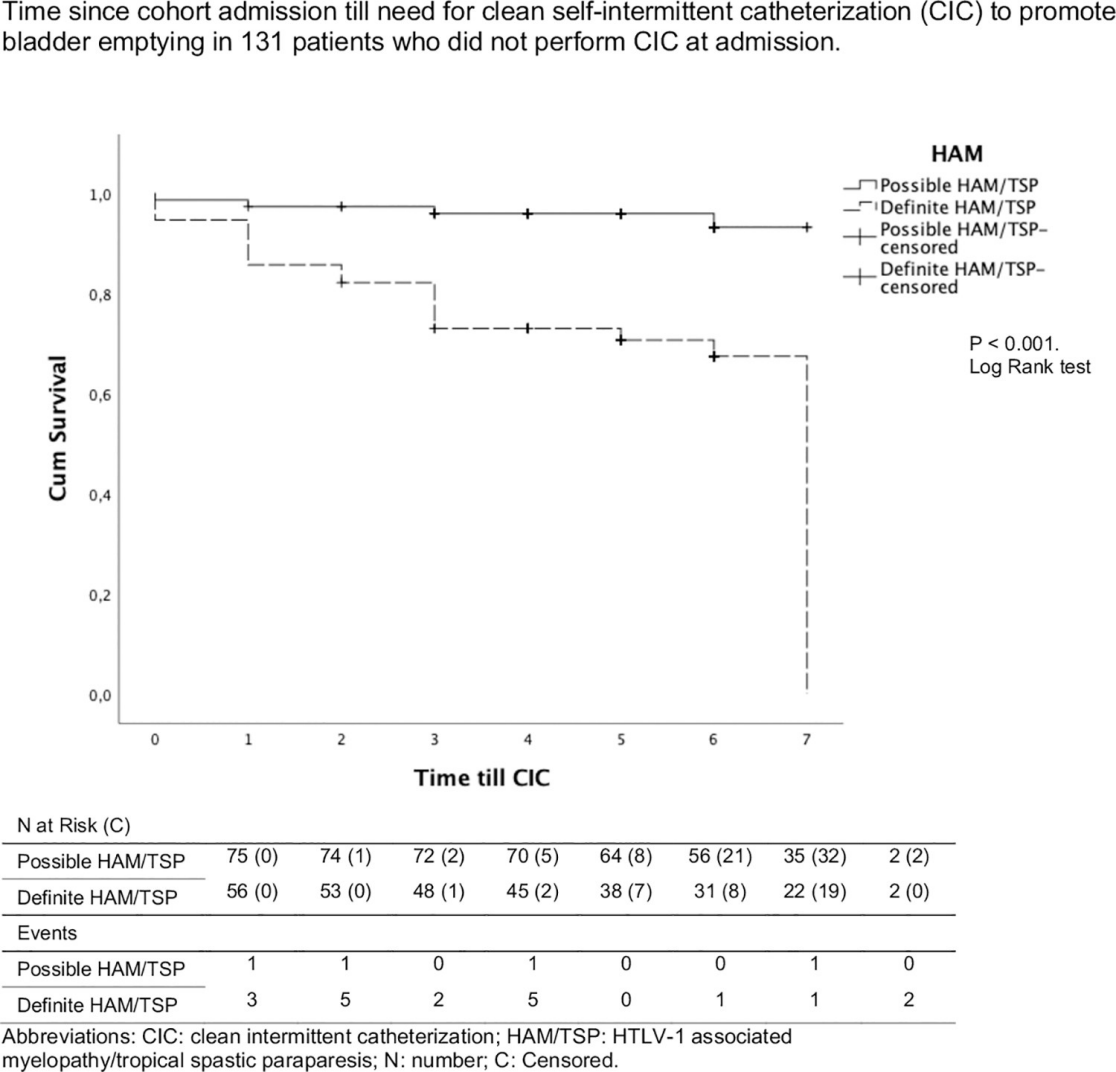
Kaplan-Meier Curves of Time to Clean Self-Intermittent Catheterization in Patients with Possible and Definite HAM/TSP. Time since cohort admission until need for clean self-intermittent catheterization (CIC) to promote bladder emptying in 131 patients free of CIC upon cohort entry.

Data regarding the absolute frequency of CIC upon study inclusion and during follow-up are shown in **Fig 1**. In the possible HAM/TSP group, two patients required CIC at baseline, and four additional patients required CIC over time. Of these four patients who experienced urological disease progression, two also presented worsening neurological symptoms and progressed from possible HAM/TSP to definite HAM/TSP during follow up, as documented by clinical findings and OSAME results; of the possible HAM/TSP patients that did not experience urological disease progression, none progressed to definite HAM/TSP during follow up. Among the definite HAM/TSP patients, 28 required CIC at baseline and 16 additional patients necessitated CIC during follow-up. Kidney injury detected by increase in the creatinine levels and attributed to urinary retention was observed in 3 patients during follow-up. They recovered renal function after foley catheter insertion followed by a switch to CIC.

Kaplan-Meier survival analysis was performed to compare time since cohort admission until need for CIC in both possible and definite HAM/TSP groups. For this analysis, only patients who were free of CIC at baseline were considered. The mean estimated time to CIC was significantly shorter in the definite HAM/TSP group. As shown in **Fig 1**, the mean estimated time to CIC was 5.5 (95% CI 4.8–6.1) years in the definite HAM/TSP group compared to 6.7 (95% CI 6.5–7.0) years in the possible HAM/TSP group (P <0.001).

## Discussion

Recent studies have shown that a large percentage of HTLV-1-infected subjects who do not meet the criteria for definite HAM/TSP suffer from symptoms or diseases related to this viral infection [8,9,27–29]. Spinal cord injury is the main cause of bladder dysfunction in patients with definite HAM/TSP [30]. However, a high percentage of patients without definite HAM/TSP also present urinary complaints characterized mainly by nocturia, urgency, incontinence and a feeling of incomplete bladder emptying [11]. As some HTLV-1 infected patients with definite HAM/TSP initially develop urinary dysfunction prior to presenting neurological symptoms and signs of myelopathy, it has been suggested that OAB or any other bladder disorder may represent an earlier stage of HAM/TSP [19,31,32]. Nonetheless, the lack of prospective studies evaluating urinary dysfunction in HTLV-1 precludes drawing definitive conclusions.

Herein, we showed that LUTS was a common finding in patients with possible and definite HAM/TSP, yet those with definite HAM/TSP exhibited higher basal frequencies of DUF and nocturia, as well as elevated OABSS values and greater need for CIC. Moreover, urodynamic studies revealed that patients with definite HAM/TSP presented not only storage symptoms, but also a higher frequency of urinary retention. These findings indicate that patients with definite HAM/TSP exhibit more exacerbated bladder dysfunction, mainly regarding the voiding function of the bladder. It follows that definite HAM/TSP patients may face a greater risk of upper urinary tract injury. Nonetheless, minor differences were seen in the other urodynamic parameters evaluated between the groups, indicating that urological dysfunction occurs similarly in individuals with possible and definite HAM/TSP.

We hypothesized that the symptoms characteristic of urinary disorders associated with HTLV-1 infection would develop gradually over time, progressing from nocturia to increased DUF, urinary urgency, incontinence and bladder areflexia [19]. Indeed, the number of patients requiring CIC to promote bladder emptying increased during follow-up in both groups, more notably in patients with definite HAM/TSP. Definite HAM/TSP patients exhibit a longstanding and robust inflammatory immune response that may lead to damage in major neural branches, provoking more dramatic consequences, such as loss of voiding function and requiring more invasive treatment, as demonstrated previously by our group [33]. Although the progressive course of voiding dysfunction of patients with definite HAM/TSP had been previously described [21,34], the present cohort extends these findings to patients with possible HAM/TSP.

Findings from urodynamic studies in patients with possible and definite HAM/TSP have been previously reported [22,35]. Herein, our evaluation of a substantial number of individuals revealed similarities in urodynamic findings between these two patient groups, with more marked urinary dysfunction noted in the definitive HAM/TSP group. Thoracic spinal cord involvement with inflammation and the subsequent narrowing of the spinal cord observed in patients with definite HAM/TSP can help explain the motor disability and high frequency of urinary dysfunction observed in these patients. However, our findings indicate that bladder dysfunction precedes motor disability in patients who do not fulfill the criteria for definite HAM/TSP.

Our study presents some limitations, such as the absence of a control group (non-infected patients), the lack of stratification or classification of overactive bladder (dry, wet and/or refractory) and lack of a formal evaluation of clinical variables that may mimic overactive bladder, such as obesity, exercise and drugs (e.g. diuretics). However, our work does offer a detailed description of HTLV-1-associated bladder dysfunction, with well-documented basal urodynamic data, in a robust sample that includes patients diagnosed with possible and definite HAM/TSP.

In summary, both groups showed similarities in bladder dysfunction and tended to progress to requiring CIC over time; however, patients with possible HAM/TSP presented less severe manifestations at baseline and progressed more slowly than those with definite HAM/TSP. Two out of four patients with progression of bladder symptoms also progressed from possible to definite HAM/TSP. Future studies focused on spinal cord findings in these patients could aid in clarifying the pathophysiology of bladder dysfunction.
